# Conservative Management of External Replacement Resorption in a Posterior Tooth: A Case Report

**DOI:** 10.7759/cureus.95474

**Published:** 2025-10-26

**Authors:** Merin Alphonsa Johnson, Asha Joseph, Anju Varughese, Sapna C Muddappa, Rakesh R Rajan

**Affiliations:** 1 Department of Conservative Dentistry and Endodontics, Amrita Vishwa Vidyapeetham, Amrita School of Dentistry, Kochi, IND

**Keywords:** ankylosis, bio ceramic sealer, cone beam computed tomography, external replacement resorption, mineral trioxide aggregate, tooth resorption management, traumatic dental injury

## Abstract

External replacement resorption (ERR) is an uncommon yet aggressive form of root resorption in which bone replaces the root following damage to the periodontal ligament. Early recognition is critical, as progression is typically silent and may ultimately result in tooth loss. This report describes a 25-year-old male patient who presented with a “sunken” lower right first molar teeth three years after biting a hard object. Clinical examination revealed infra-occlusion, a high-pitched metallic percussion tone, and delayed pulp responses. Periapical radiography and cone beam computed tomography demonstrated loss of periodontal ligament space and trabecular bone within the distal root, confirming progressive ERR with pulpal necrosis and asymptomatic apical periodontitis. Conservative non-surgical root canal therapy was undertaken, during which calcium hydroxide was placed as an intracanal medicament for four weeks to suppress clastic activity. Subsequently, the distal canal was obturated incrementally with mineral trioxide aggregate, while the mesial canals were filled with gutta-percha and a calcium-silicate bioceramic sealer. A composite core was built, and a computer-aided design and computer-aided manufacturing (CAD/CAM)-milled zirconia crown was cemented to restore occlusion. Follow-up at six and twelve months demonstrated resolution of symptoms, stable occlusion, and radiographic evidence of periapical healing without further resorptive progression. Early diagnosis aided by cone beam computed tomography (CBCT) and a biologically based, calcium-silicate obturation approach allowed successful preservation of a mandibular molar affected by progressive ERR, highlighting that conservative endodontic management with bioactive materials can be a predictable option even in advanced cases.

## Introduction

External replacement resorption (ERR), or ankylosis, presents a complex diagnostic and therapeutic challenge, especially when the lesion progresses undetected. Root resorption refers to the gradual degradation of cementum and dentine due to the activity of clastic cells [1]. Various etiological factors of tooth root resorption are mentioned in the literature, among which trauma, inflammation and iatrogenic factors, such as bleaching and orthodontic treatment, are especially noteworthy. Regardless of the various causative factors, the etiology of some types of resorption remains unclear, necessitating further research [2]. ERR is a distinct type of external root resorption where bone progressively replaces the root structure, often leading to eventual loss of the affected tooth [3]. Its asymptomatic onset emphasizes the importance of early identification through vigilant clinical and radiographic examination [1].

Although relatively rare in practice, root resorption is usually prevented by several biological mechanisms, such as the intact periodontal ligament (PDL), cementum layer, and extracellular matrix components like predentin [4]. Osteoclasts, the large, multinucleated cells, play the central role in resorbing mineralized tissues [5]. Unlike external inflammatory root resorption (EIR), which typically follows infection, ERR may be seen as a physiological process of skeletal remodeling once ankylosis has set in [6]. Over time, bone trabeculae proliferate within the periodontal space and integrate with the root surface [7]. Initially, this may involve the formation of bone bridges that eventually give rise to further cellular infiltration and root replacement [8].

Management strategies range from decoronation and orthodontic space closure to extraction followed by bone grafting and implant rehabilitation [9]. A recent literature by Thakur et al. has demonstrated the use of a bioceramic material for management of invasive external root resorption in an anterior tooth using a bioceramic material and have shown successful healing [10]. However, there remains some debate regarding optimal approaches, particularly for posterior teeth [11]. There is limited literature available on the management of external replacement resorption, as its treatment remains particularly challenging due to the progressive nature of the condition and the replacement of root structure by bone. The following report discusses the successful non-surgical management of ERR in a mandibular first molar using calcium silicate-based root filling and prosthetic rehabilitation with a zirconia crown.

## Case presentation

A 25-year-old male presented to the Department of Conservative Dentistry and Endodontics with a complaint of a sunken lower right posterior tooth. The patient reported that the issue began approximately three years prior, following trauma from biting on a hard object. Initially, discomfort and pain were present but gradually resolved without intervention.

**Figure 1:**
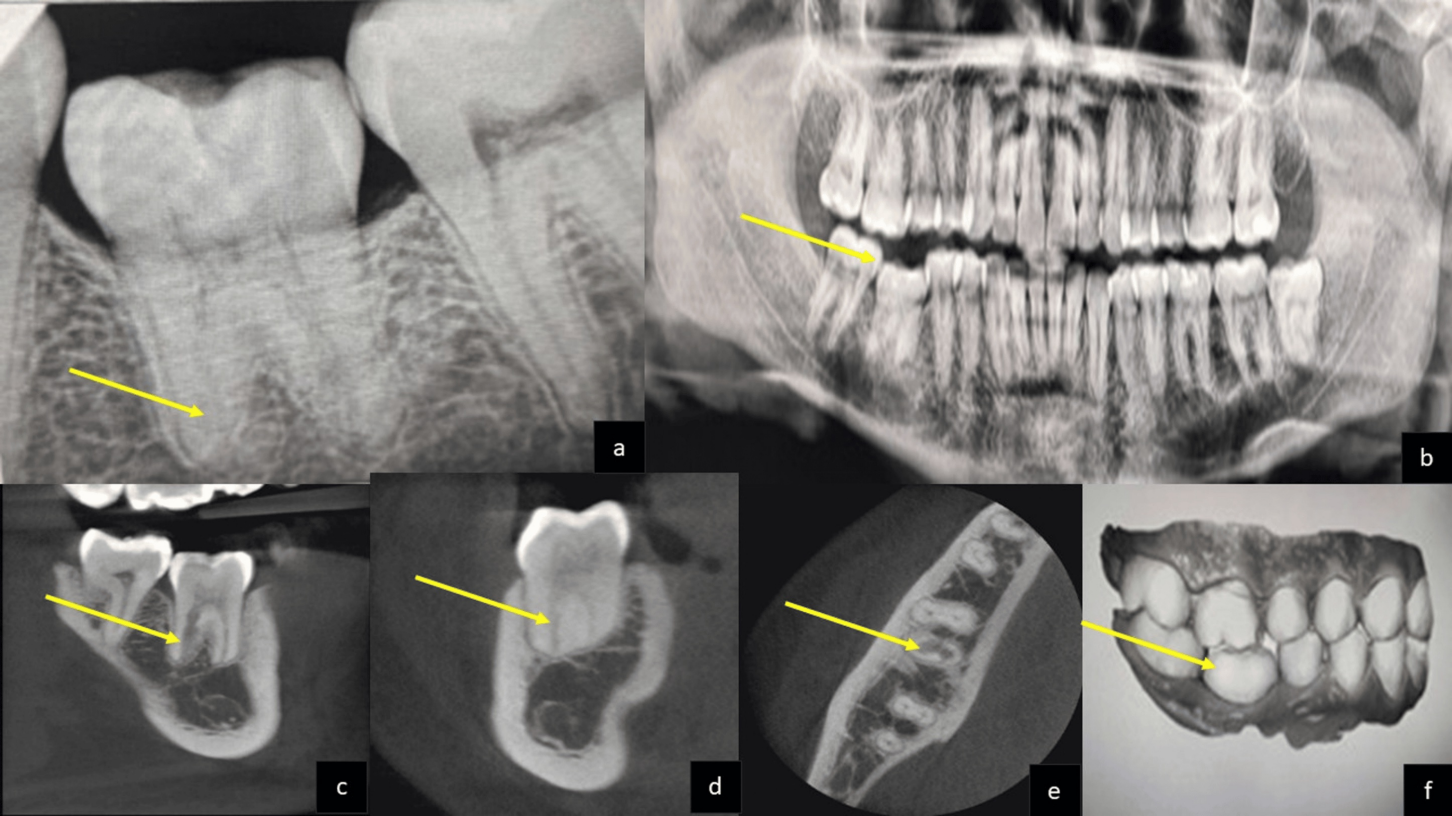
(a) Pre-operative RVG shows indistinct distal root outline and absence of lamina dura, (b) pre-operative OPG-submerged 46, 16 supraerupted, (c) pre-operative CBCT sagittal view, (d) coronal view, (e) axial view, and (f) pre-operative intraoral scanning image-approximately 0.5 mm space can be appreciated between the two molars. RVG: radiovisiography; OPG: orthopantogram; CBCT: cone beam computed tomography.

Intraoral examination revealed infra-occlusion of tooth, mandibular right first molar (#46), which responded with a high-pitched, metallic sound on percussion testing, though it was not tender. Maxillary right first molar (#16) showed supra-eruption and slight chipping of the mesial enamel. Pulp sensibility testing of tooth #46 showed delayed responses to both thermal and electric stimuli. Digital periapical radiographs showed an indistinct root outline of the distal root of tooth #46 (Figure 1a). Orthopantogram (OPG) shows #46 in an infra-occlusal position (Figure 1b). Cone beam computed tomography (CBCT) imaging was employed to further evaluate the lesion. The scan demonstrated irregular trabecular bone within the distal root, complete absence of the PDL space, and partial calcification of the pulp chamber (Figures 1c-1e). A digital scan confirmed limited occlusal clearance (0.5 mm) between teeth #16 and #46 (Figure 1f). Based on clinical and radiographic assessments, a diagnosis of pulpal necrosis with asymptomatic apical periodontitis associated with tooth #46 was made. The treatment plan involved non-surgical root canal therapy, followed by definitive occlusal restoration.

At the initial visit, endodontic access was established under local anesthesia (Lignospan, Septodont, Saint-Maur-des-Fossés, France). Working lengths were determined using a No. 15 stainless steel hand K-file (M-access; Dentsply Maillefer, Ballaigues, Switzerland) as 16 mm for the mesiobuccal (MB), 16.5 mm for the mesiolingual (ML), and 17 mm for the distal canal. The distal canal exhibited profuse bleeding and granulation tissue. Cleaning and shaping were performed up to size 30, 4% taper for MB and ML canals, and size 30, 6% taper for the distal canal using the Hanu Dent® rotary file system (Hanu Dent Pvt. Ltd., Vattiyoorkavu, India). During instrumentation, 30-gauge side-vented needle irrigation (Dentsply Sirona Inc., Charlotte, NC) was carried out with 1.5% sodium hypochlorite (NaOCl) (Prime Dental, Thane, Maharashtra, India) (10-15 ml per canal; 10-15 minutes cumulative exposure); intermittent irrigation was done with 17% ethylenediaminetetraacetic acid (EDTA) liquid (Smartprep; SafeEndo, Vadodara, Gujarat, India) (1-2 mL per application, one to two minute contact time per application); and with a final flush of 2% chlorhexidine (CHX, HexaChlor, SafeEndo, Vadodara, Gujarat, India) (1-2 mL of 2% CHX per canal, contact time of one minute) and saline (2-3 mL per canal) [12]. Calcium hydroxide with iodoform (Meta Biomed Metapex, Meta Biomed Co., Ltd., Cheongju, South Korea) was applied as an intracanal medicament and left in place for one month to promote disinfection and reduce osteoclastic activity [13,14].

**Figure 2:**
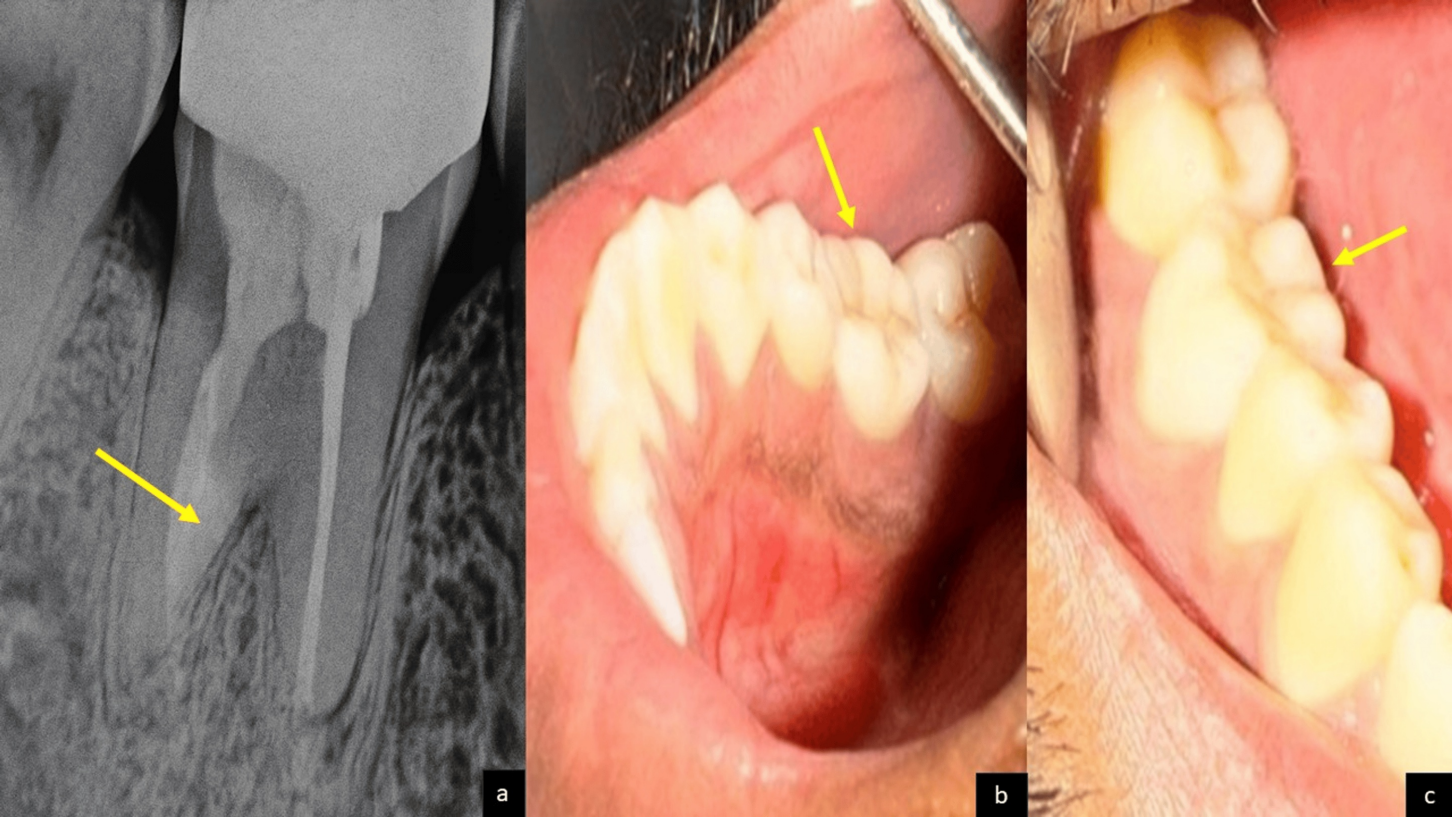
(a) Immediate post-operative radiograph (RVG), (b) immediate post-operative intraoral photograph from lingual view, and (c) immediate post-operative intraoral photograph from buccal view. RVG: radiovisiography.

**Figure 3:**
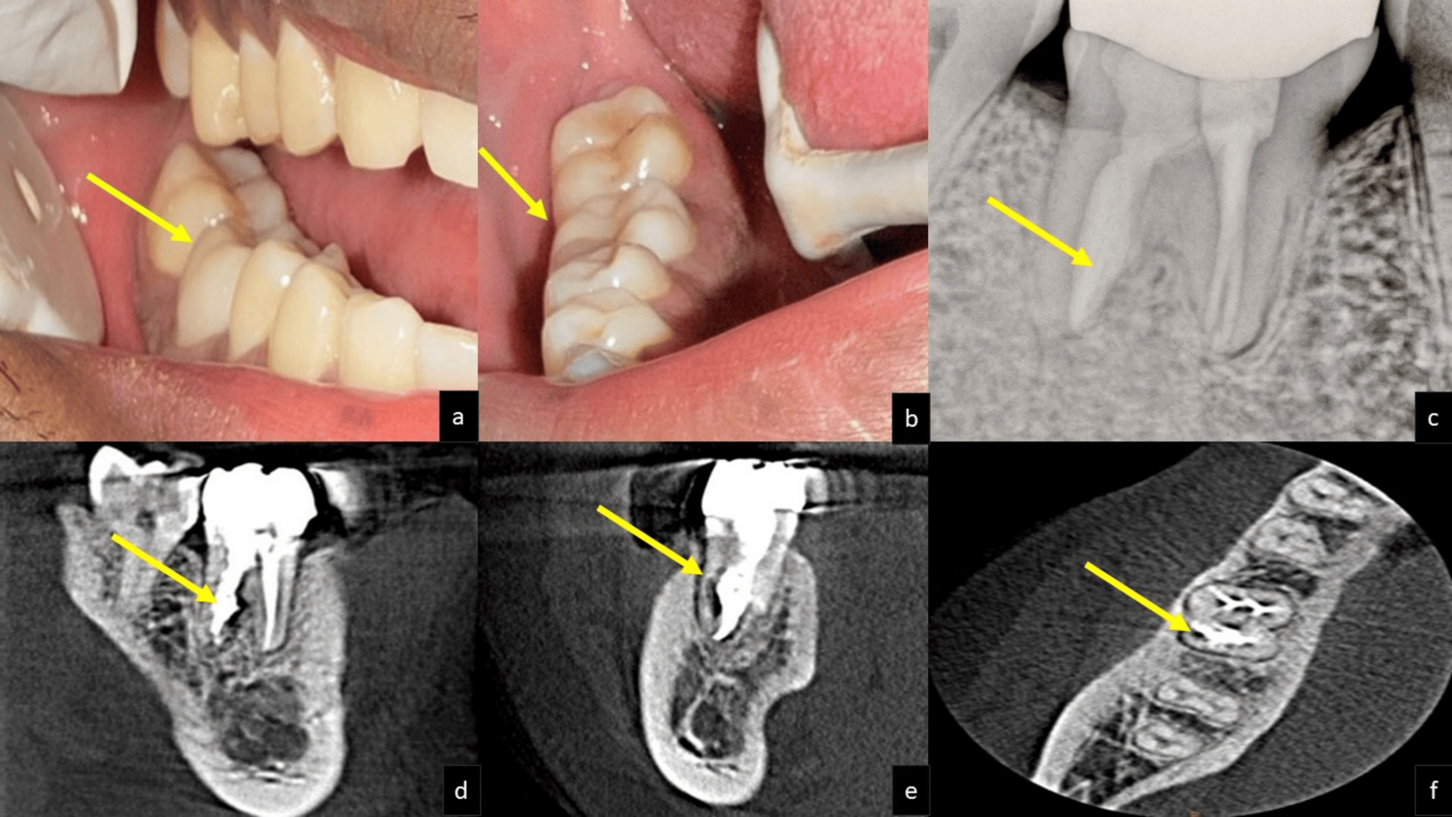
On a 12-month review. (a) Intraoral buccal view, (b) intraoral lingual view, (c) intraoral periapical radiograph (RVG), (d) CBCT-sagittal view, (e) coronal view, and (f) axial view. CBCT: cone beam computed tomography; RVG: radiovisiography.

At the follow-up appointment, the medicament was removed using H-files and copious irrigation with saline. The distal canal was obturated incrementally using mineral trioxide aggregate (MTA) (MTA Angelus®, Angelus Indústria de Produtos Odontológicos S/A, Londrina, Brazil) via a Messing gun. The mesial canals were filled with gutta-percha cones and BioRoot™ RCS, a calcium silicate-based sealer. Composite (3M™ Filtek™ Z250XT Nano Hybrid Universal Restorative A3, Syringe, 3M ESPE, St. Paul, Minnesota) was placed for core build-up. To restore occlusal function and prevent further trauma, a computer-aided design and computer-aided manufacturing (CAD/CAM)-fabricated zirconia crown was cemented (Figures 2a-2c) after a one-week review. The patient was monitored after six months and 12 months, with follow-ups showing maintained function, good periapical healing, and no evidence of ongoing resorption (Figures 3a-3f). At the 12-month follow-up, a CBCT was taken to evaluate the rate of healing at the resorptive site. Comparative evaluation of pre-operative and 12-month post-operative CBCT scans revealed measurable changes suggestive of stabilization of external replacement resorption. The resorptive area on the mesial aspect of the distal root showed a reduction in size from approximately 5.4 mm² to 3.1 mm², corresponding to an estimated decrease of nearly 40% in the resorption defect volume. The overall root length, measured from the cementoenamel junction to the apex, remained unchanged (approximately 13.6 mm), indicating arrest of further root resorption. Overall, the CBCT findings indicated arrested progression of replacement resorption and improvement in periradicular bone density, signifying successful control of resorptive activity following disinfection and bioceramic barrier placement.

## Discussion

External replacement resorption is a pathological condition marked by root replacement with bone following the loss of protective periodontal structures. The condition typically develops due to trauma, mechanical stress, or infection that damages cementoblasts and the precementum [15]. In contrast to alveolar bone, permanent teeth do not undergo physiological resorption. Once initiated, ERR is challenging to reverse and may lead to early tooth loss if not addressed [13]. The subtle and varied clinical presentation of ERR makes diagnosis difficult. In this case, infra-occlusal metallic percussion sound and a delayed pulp response suggested ankylosis, which was confirmed radiographically. CBCT played a vital role in identifying the absence of the PDL and replacement of the root by bone-like structures, consistent with ERR [3].

Although replacement resorption may present clinically with a high-pitched metallic percussion sound and loss of mobility, patients often report no symptoms. In growing individuals, such teeth may appear infra-occluded [16]. This suggests that unnoticed trauma during the eruptive period, possibly in childhood, may have contributed to the current pathology, despite the patient only recalling a more recent episode. Calcium hydroxide has long been used to manage root resorption due to its high pH and ability to modulate clastic cell activity [17].

In this clinical scenario, MTA was used for obturating the resorbed distal root. Its superior biocompatibility, sealing ability, and osteogenic potential make it an ideal material for irregular resorption cavities [16]. Gutta-percha and a bioceramic sealer were used for the structurally intact mesial canals. The choice of a zirconia crown ensured strength, occlusal stability, and long-term durability. The most common approach for the management of external replacement resorption is surgical intervention. But it is often associated with limited long-term success, progressive alveolar bone loss, and esthetic concerns, particularly in young patients, making it a less favorable option compared to the conservative approach. This highlights the importance of minimally invasive management for external replacement resorption [18].

## Conclusions

At regular follow-up intervals, the tooth remained asymptomatic, functional, and free of further resorptive changes. It was confirmed with a 12-month review of CBCT. This shows healing of the resorptive lesion and the absence of further progression. A conservative management approach helped maintain the tooth without a surgical intervention. This conservative management of external replacement resorption was found to be efficient in arresting the resorptive lesion; therefore, the treatment can be considered clinically successful, and further follow-ups are required at yearly intervals.
